# Impact of immediate initiation of antiretroviral therapy among men who have sex with men infected with HIV in Chengdu, southwest China: trends analysis, 2008–2018

**DOI:** 10.1186/s12889-021-10580-8

**Published:** 2021-04-08

**Authors:** Chenyao Wu, Baiyang Zhang, Zhen Dai, Qianwen Zheng, Zhenhua Duan, Qinying He, Cairong Zhu

**Affiliations:** 1 Department of Epidemiology and Health Statistics, West China School of Public Health and West China Fourth Hospital, Sichuan University, 610041 No. 17 Section 3, Renmin South Road, Chengdu, Sichuan China; 2 Department of AIDS&STD Control and Prevention, Chengdu Center for Disease Control and Prevention, 610041 No. 4 Longxiang Road, Sichuan Chengdu, China

**Keywords:** HIV infections, Homosexuality, male, Antiretroviral therapy, highly active, HIV seroprevalence, Health policy, Interrupted time series analysis

## Abstract

**Background:**

Given the rampant HIV epidemic among men who have sex with men (MSM) in Chengdu, southwest China, Treat All policy, defined as immediate antiretroviral therapy (ART) initiation after HIV diagnosis, was implemented since 2014. Real-world research evaluating impacts of immediate ART on HIV epidemics is needed to optimize policy-making as national and international guidelines have been lowering ART eligibility threshold. The purpose of this study is to: assess temporal trends of the HIV epidemic and impacts of Treat All policy among MSM; and lay foundation for HIV-related policy evaluation using longitudinal routine data from health information systems.

**Methods:**

Data used in this study were HIV sentinel seroprevalence, annual reported HIV cases and ART coverage rate among MSM in Chengdu from 2008 to 2018, derived from national HIV/AIDS information system. Temporal trends of the HIV epidemic were described using Joinpoint Regression Program. Interrupted time-series method was deployed to evaluate Treat All policy.

**Results:**

HIV sentinel seroprevalence rose from 11.20% in 2008 to 17.67% in 2013 and Annual Percent Change (APC) was 8.25% (95% CI − 2.40%, 20.07%), then decreased to 5.17% in 2018 (APC = − 19.63%, 95% CI − 27.54%, − 10.86%). Newly reported HIV cases increased from 168 cases in 2008 to 1232 cases in 2015 (APC = 26.99%, 95% CI 21.32%, 32.93%), and reduced to 1014 cases in 2018 (APC = − 8.80%, 95% CI − 18.45%, 2.01%). ART coverage rate has been climbing from 11.11% in 2008 to 92.29% in 2018 and Average Annual Percent Change was 16.09% (95% CI 11.76%, 20.59%). Results of interrupted time-series models showed that compared to an annual increase of 0.87% during pre-policy period, there was a decline of 3.08% (95% CI − 0.0366%, − 0.0250%) per year of HIV sentinel seroprevalence since 2014; and compared to an annual increase of 116 cases before 2014, there was an annual drop of 158 newly reported HIV cases (95% CI − 194.87%, − 121.69%) during the post-policy period.

**Conclusions:**

Immediate ART after HIV diagnosis could potentially curb HIV transmission at population level among MSM, along with other strategies. Future assessment of HIV prevention and control policy can be carried out using routinely collected longitudinal data from health information systems.

## Background

The HIV pandemic continues to be a major global public health issue. World Health Organization (WHO) estimated there were approximately 37.9 million people living with HIV (PLHIV) by the end of 2018 [1]. The distribution of China’s HIV epidemics is unbalanced by geographic region and subpopulation group. Both prevalence and incidence of HIV are especially high in southwest China, and men who have sex with men (MSM) bear a disproportionate burden of HIV [2], accounting for 25.5% of national new HIV infections in 2018, rising from 2.5% in 2006 [3, 4].

MSM, whose risk of acquiring HIV is 22 times higher than the general population, are listed as key population in the HIV pandemic by the Joint United Nations Program on HIV and AIDS (UNAIDS) [5]. Preventive measures such as condom use help contain HIV infection [6], but they don’t reduce infectiousness (defined by plasm viral load) or HIV-related morbidity for PLHIV. Whilst antiretroviral therapy (ART) can optimize health outcomes for PLHIV and reduce onward HIV transmission by lowering plasm viral loads [7–9]. Specially, starting ART at early stages of infection, compared with a deferred initiation, has been proved to have individual-level benefits, including less morbidity and mortality and reduced rate of linked partner transmissions [10–17]. Based on clinical significance of immediate ART after HIV diagnosis and development of new antiretroviral drugs with fewer treatment-related toxic effects [10, 12, 17–22], CD4+ cell threshold eligible for ART in both national and international guidelines for HIV treatment has been relaxed to shorten time from diagnosis to ART in recent years [23, 24]. Nevertheless, several randomized controlled trials found that HIV incidence in the study population wasn’t substantially reduced in the context of immediate ART for all PLHIV [25–27]. Despite the individual-level benefits, impacts of immediate ART on HIV infection at population level is unknown. Mathematic models predicted that, among MSM, early ART might reduce HIV incidence and prevalence [28, 29], pending further empiric evidence (we used early ART, immediate ART and Treat All interchangeably). Real-world research has been listed as high priority area in MSM HIV prevention and control [30], whereas there are limited researches evaluating the effects of HIV policies targeted at MSM in a real-world setting. Real-world policy evaluation is necessary to provide empiric evidence for policy makers and health care workers. Hence real-world researches in MSM HIV epidemics are urgently needed, given the especially high HIV incidence among MSM, to mitigate the knowledge gap and facilitate policy making in MSM HIV prevention and control [30].

Sichuan province, the most populous and developed region in southwest China, has ranked first nationally with annual reported HIV cases for years [31]. The provincial capital of Sichuan province, Chengdu, is one of the five urban regions in China where HIV is spreading at an alarming rate with substantial increases in MSM population [2]. Given the raging HIV epidemic among MSM in Chengdu [2], the local Center for Disease Control and Prevention (CDC) determined that Treat All policy be implemented among MSM since 2014; under this policy, MSM can receive free ART immediately after HIV diagnosis regardless of CD4+ cell count. Treat All policy began two years earlier prior to the 2016 national and WHO guidelines recommending ART be offered to all HIV-positive adults and adolescents, irrespective of CD4+ cell count. With national and international guidelines lowering ART threshold, assessment of Treat All policy should be performed since its implementation, to shed a light on whether “treat all” helps to contain the HIV epidemic and set an example for studies of this kind.

The purpose of this study, therefore, is to assess temporal trends of the HIV epidemic and ART coverage among MSM in Chengdu before and after Treat All policy; to evaluate impacts of Treat All policy on MSM HIV infection at population level; and to lay foundation for HIV policy evaluation using longitudinal administrative data, as well as provide evidence for policy makers to further improve early HIV treatment outcomes.

## Methods

### Study design

This is a retrospective study using longitudinal and routinely collected data from national health information system between 2008 and 2018.

### Data source and indicators

Indicators analyzed in this study were: HIV sentinel seroprevalence, newly reported HIV cases and ART coverage rate among MSM whose address was Chengdu city. The indicators were aggregated or calculated for each year between 2008 and 2018, using data derived from National HIV/AIDS Comprehensive Response Information Management System (CRIMS). After years’ development since 1990’s, China’s web-based HIV information system was established in 2005 to promote timely reporting and data quality. With improvements and expansion of HIV/AIDS data collection, a multifaceted web-based HIV/AIDS information system called CRIMS was put into operation since 1st January 2008. To ensure data quality and completeness, there are laws regulating data collecting process, staff training, operational manual for CRIMS and quality assessments carried out annually, thereby data from CRIMS can be deemed as reliable for our analyses.

As part of CRIMS, China’s HIV surveillance comprises two main databases: (1) routine online HIV case reporting by health care providers at all levels, legally mandated by national regulations; (2) HIV sentinel surveillance annually collecting cross-sectional data including HIV serostatus in sentinel sites across China, to consecutively monitor HIV epidemic situations among key affected populations [32–35]. Chengdu is a southwest sentinel site of National HIV Sentinel Surveillance System, and MSM have been its key monitoring group since 2004. Besides, databases of HIV counseling and testing, ART management and the like are parts of CRIMS as well. More detailed information about data collection, management and quality control can be found in previous study [36].

Data from HIV surveillance (HIV seroprevalence and newly reported HIV cases) reflect trends of the HIV epidemic and have been used in many studies [2, 4, 37, 38], information from the data can be regularly applied by policy makers to optimizing HIV prevention efforts. HIV sentinel surveillance and routine HIV case reporting provide valuable and perhaps the only reliable information about HIV epidemic situations as well as evidence for HIV policy evaluation among MSM [39, 40], under the circumstance that MSM come from a hidden and stigmatized population whose size is hard to estimate [41].

#### HIV sentinel seroprevalence

HIV sentinel seroprevalence, defined as the proportion of the sampled MSM who are tested and confirmed as HIV infected in the annual sentinel survey which is conducted at fixed locations and lasts from April to June every year since 2005. It was calculated with number of sampled MSM in the annual sentinel survey conducted in Chengdu as denominator and confirmed HIV cases from those MSM as numerator.

HIV sentinel surveillance is in the charge of local CDCs designated by national health authority and executed in accordance with Operational Manual of The National AIDS Sentinel Surveillance Program. According to the Operational Manual, MSM found to be previously HIV infected should be included, and sample size depends on HIV prevalence (250 if prevalence more than 10%, 400 otherwise for MSM). The MSM population in HIV sentinel surveillance is defined as men who had oral or anal intercourse with other males within the past year. Sampling methods are as below: (1) snowball sampling; (2) recruitment from venues frequented by MSM like gay bars; (3) recruitment via networks. After informed consent is signed, 3 to 5 mL venous blood is sampled for HIV serological test and HIV serostatus is determined by corresponding technical guidance.

#### Newly reported HIV cases

Newly reported HIV cases used in this study were newly confirmed cases registered in CRIMS whose route of HIV infection was male homosexual transmission and address was Chengdu city. Annual reported HIV cases were aggregated from 2008 to 2018.

Given the hidden and stigmatized nature of MSM [39], it’s deemed that newly reported HIV cases is a marker for HIV incidence, and trends in new HIV cases have been found to be consistent with trends in estimates of HIV incidence [42–44].

#### ART coverage rate

ART coverage rate is defined as the percentage of living HIV-positive MSM registered in CRIMS who have been receiving ART. MSM in the treatment database (after receiving treatment) were linked to their own records in the epidemiology database (after confirmed as HIV positive). It was calculated with number of registered alive MSM HIV cases whose address was Chengdu city as denominator and linked cases receiving ART as numerator.

Changes of ART coverage can reflect changes in CD4+ cell count threshold for ART eligibility. Increasing coverage was found to be related to a decreasing threshold [13], thereby ART coverage can be used to directly reflect effects of the treatment eligibility threshold changes.

### Statistical methods

Temporal trends analysis was conducted with Joinpoint Regression Program (Version 4.7.0.0 - February 2019, National Cancer Institute) to depict HIV seroprevalence, newly reported HIV cases and ART coverage among MSM in Chengdu from 2008 to 2018. In this approach, Joinpoint models that best fitted the data were built and corresponding temporal trend curves were drawn, assuming the change in outcome variable is constant over each time partition defined by the transition points (called joinpoints), but varies among different time partitions. Annual Percent Change (APC) and Average Annual Percent Change (AAPC) reflect relative change of outcome variable. APC is an estimated annual percentage change where the outcomes of interest are assumed to change at a constant percentage of that of the previous year. AAPC is a summary measure of the trend to describe the average APCs over a period of multiple years.

Interrupted time-series (ITS) method was applied to evaluate impacts of Treat All policy on HIV transmission at population level among MSM [45]. By using longitudinal and routinely collected data from health systems before and after a policy intervention, ITS analysis can evaluate impacts of health policies on outcome of interest without requiring a control site. This method was based on fitting segmented linear regression model, which divided the time series into pre-policy (including 6 data points from 2008 to 2013) and post-policy (including 5 data points from 2014 to 2018) segments. Analysis was performed using Stata 14.0 (StataCorp. 2014).

The following model was established for ITS analysis [46]:

*y*_*t*_ *= β*_*0*_ *+ β*_1_*time + β_2_*policy + β_3_*postslope + ε_*t*_, where *y*_*t*_ was the outcome variable at different time points, variable *time* indicates time points and was coded number 1–11 corresponding to 2008–2018 in year. Variable *postslope* was coded 0 up to the last point before the policy intervention and coded sequentially from 1 thereafter. *β*_*0*_ estimates the baseline level of *y*_*t*_ at the beginning of the time series. *β*_*1*_ estimates the structural trend or natural growth rate of *y*_*t*_, independently from the policy intervention. *β*_*2*_ estimates the change in level of *y*_*t*_ due to policy intervention (*policy* = 0 and *policy* = 1 indicate before and after the policy intervention respectively), reflecting immediate impact of the policy. *β*_*3*_ estimates the change in trend of *y*_*t*_ after the policy intervention, reflecting the long-term effect of the policy. *β*_*2*_ and *β*_*3*_ represent absolute annual change of outcome variable. *β*_*2*_ answers whether there’s an immediate impact of, while *β*_*3*_ answers whether there’s a long-term or sustained impact of the policy intervention. *ε*_*t*_ is the random error at time points [46].

Our outcomes of interest were HIV sentinel seroprevalence and annual reported HIV cases, a visual examination was used to assess the trend or non-stationary characteristics of the data. Then possible autocorrelation between values at serial time points was assessed using the Durbin-Watson test, where both outcome variables showed autocorrelation. Hence generalized least squares estimator was applied, using Prais-Winston method to correct data autocorrelation [47].

The overall significance level was *ɑ* = 0.05.

## Results

### HIV sentinel seroprevalence and temporal trend

**Fig. 1:**
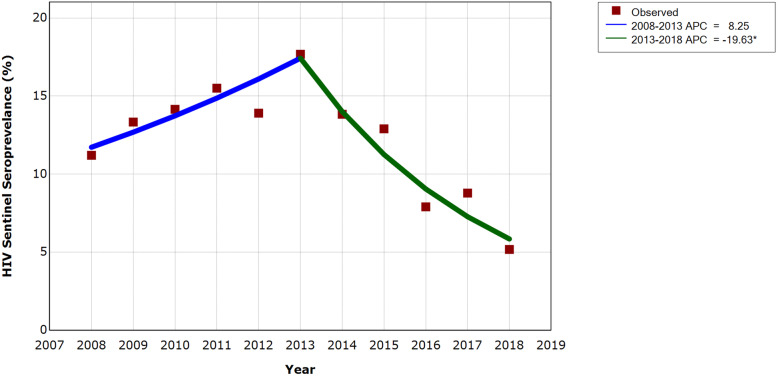
Temporal trend of HIV sentinel seroprevalence among MSM in Chengdu between 2008 and 2018. *Indicates that the Annual Percent Change (APC) is significantly different from zero at the alpha = 0.05 level. Joinpoint: year 2013

HIV sentinel seroprevalence among MSM in Chengdu showed a rising trend from 11.20% in 2008 to 17.67% in 2013, then dropped all the way to 5.17% in 2018 (Table 1). Annual Percent Change (APC) between 2008 and 2013 was 8.25% (95% CI − 2.40%, 20.07%), which wasn’t statistically significant, entailing uncertainty about the change direction of the trend before 2014. The significantly negative APC of 2013–2018 (− 19.63%, 95% CI − 27.54%, − 10.86%) reflected a downward trend of HIV seroprevalence after Treat All policy intervention (Fig. 1). Besides, Average Annual Percent Change (AAPC) of 2008–2018 (− 6.72%, 95% CI − 12.04%, − 1.09%) was significantly below zero, indicating an overall declining trend over the observation period.

**Table 1:** HIV sentinel seroprevalence, newly reported HIV cases and ART coverage rate among MSM in Chengdu between 2008 and 2018

	2008	2009	2010	2011	2012	2013	2014	2015	2016	2017	2018
**HIV sentinel seroprevalence (%)** ^**a**^	11.20	13.33	14.15	15.50	13.90	17.67	13.83	12.90	7.90	8.78	5.17
**Newly reported HIV cases** ^**b**^	168	340	367	528	639	824	1061	1232	1124	1021	1014
**ART coverage rate (%)** ^**c**^	11.11	14.56	25.71	40.24	47.16	57.51	65.57	78.40	85.21	90.21	92.29

^a^ calculated with number of sampled MSM in the annual sentinel survey conducted in Chengdu as denominator and confirmed HIV cases from those MSM as numerator

^b^ newly confirmed HIV cases in CRIMS whose route of infection was male homosexual transmission and address was Chengdu city

^c^ calculated with number of alive MSM HIV cases registered in CRIMS whose address was Chengdu city as denominator and linked cases receiving ART as numerator

### Newly reported HIV cases and temporal trend

**Fig. 2:**
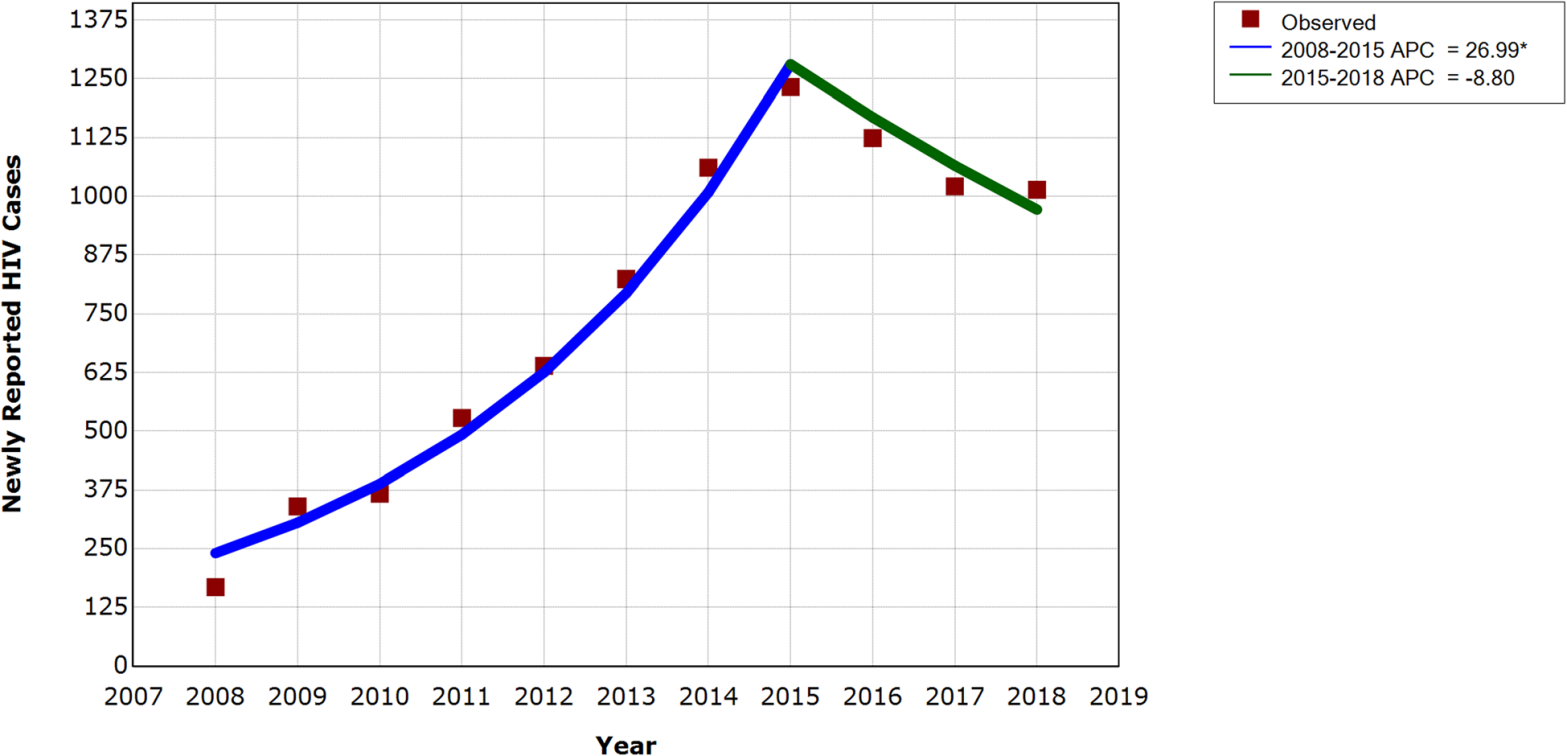
Temporal trend of newly reported HIV cases among MSM in Chengdu between 2008 and 2018. *Indicates that the Annual Percent Change (APC) is significantly different from zero at the alpha = 0.05 level. Joinpoint: year 2015

Newly reported HIV cases among MSM in Chengdu rapidly increased from 168 cases in 2008 to 1232 cases in 2015, then decreased to 1014 cases in 2018, remaining at a relatively high level during post-policy period compared with that in earlier years (Table 1). APC was 26.99% (95% CI 21.32%, 32.93%) between 2008 and 2015, suggesting a significant upward trend during this period. APC of 2015 to 2018 was − 8.80% (95% CI − 18.45%, 2.01%), nonsignificant and entailing uncertainty about the change direction of the trend after 2014 (Fig. 2). While AAPC over 2008 to 2018 was 14.99% (95% CI 10.79%, 19.34%), with an upper 95% CI limit smaller than the lower 95% CI limit of the APC of 2008 to 2015, indicating a slower growing rate on average over 2008 to 2018 than that during 2008 to 2015.

### ART coverage rate and temporal trend

**Fig. 3:**
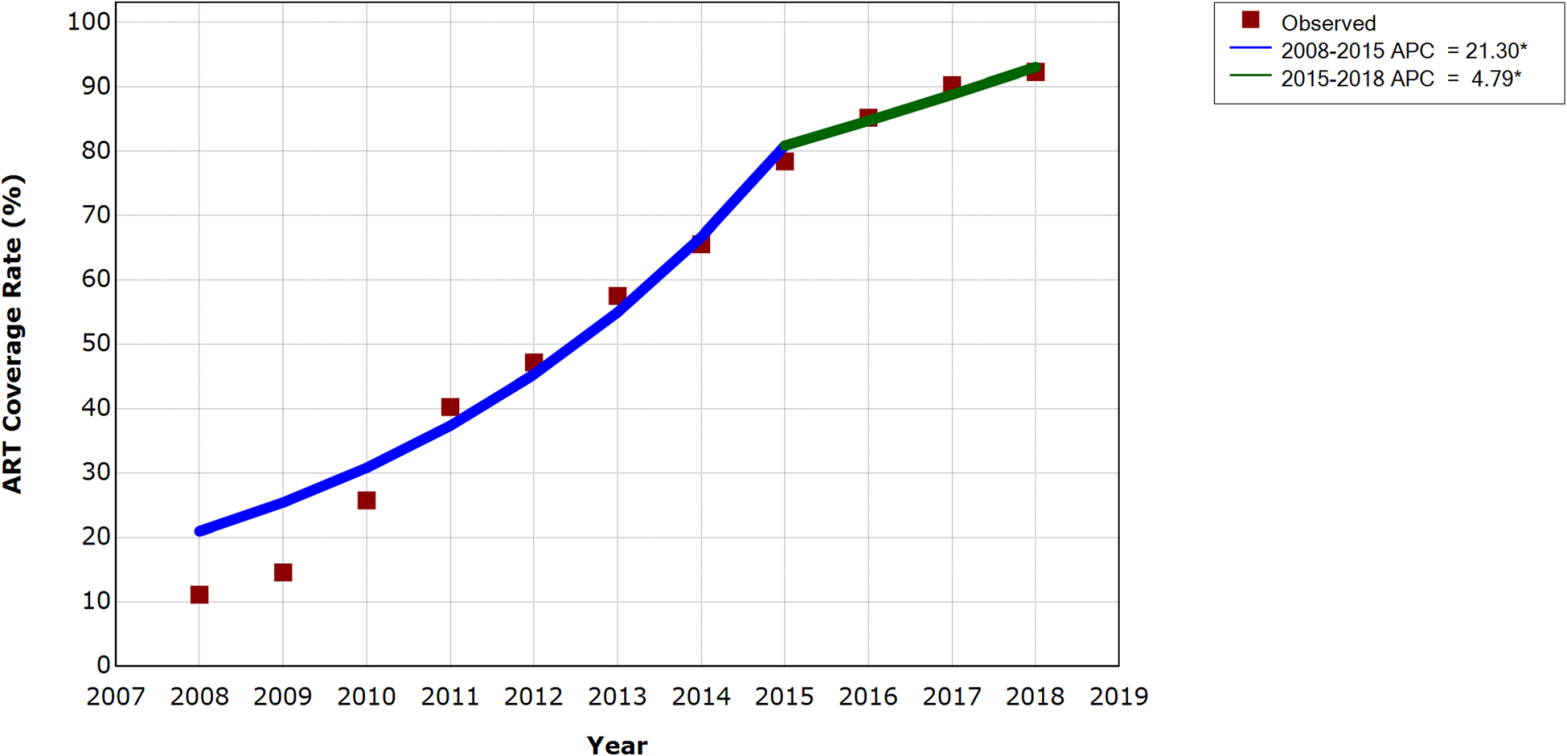
Temporal trend of ART coverage rate among MSM in Chengdu between 2008 and 2018. *Indicates that the Annual Percent Change (APC) is significantly different from zero at the alpha = 0.05 level. Joinpoint: year 2015

ART coverage rate among MSM in Chengdu has been climbing over the span of 2008 to 2018: from 11.11% in 2008 to 92.29% in 2018 (Table 1). APC between 2008 and 2015 (21.30%, 95% CI 13.45%, 29.68%) was higher than that of 2015 to 2018 (APC = 4.79%, 95% CI 2.03%, 7.62%), implying ART coverage rate grew quickly prior to 2015 and then slightly increased, gradually stabilizing at a high level ever since Treat All policy was implemented (Fig. 3).

### Interrupted time-series analysis

Table 2 displays the results of segmented linear regression model 1 and 2 built to evaluate the impacts of Treat All policy on HIV sentinel seroprevalence and annual reported HIV cases respectively among MSM in Chengdu.

**Table 2:** Results of segmented regression models for HIV sentinel seroprevalence and annual reported HIV cases among MSM before and after Treat All policy

	Coefficient	Standard error	***P*** value	95% Confidence interval (CI)
**HIV sentinel seroprevalence** ^**a**^ **(Model 1)**				
*β*_*0*_ (Baseline)	0.1123	0.0059	< 0.001***	(0.0982, 0.1264)
*β*_*1*_ (Structural trend)	0.0087	0.0015	0.001***	(0.0051, 0.0124)
*β*_*2*_ (Change in level)	− 0.0005	0.0087	0.959	(− 0.0210, 0.0201)
*β*_*3*_ (Change in trend)	− 0.0308	0.0025	< 0.001***	(− 0.0366, − 0.0250)
**Newly reported HIV cases** ^**b**^ **(Model 2)**				
*β*_*0*_ (Baseline)	63.35	37.33	0.133	(−24.92, 151.62)
*β*_*1*_ (Structural trend)	116.38	9.68	< 0.001***	(93.48, 139.28)
*β*_*2*_ (Change in level)	463.23	54.65	< 0.001***	(334.01, 592.45)
*β*_*3*_ (Change in trend)	−158.28	15.47	< 0.001***	(−194.87, −121.69)

^a^ calculated with number of sampled MSM in the annual sentinel survey conducted in Chengdu as denominator and confirmed HIV cases from those MSM as numerator

^b^ newly confirmed HIV cases in CRIMS whose route of infection was male homosexual transmission and address was Chengdu city

Results of Model 1(Table 2) demonstrated that no significant change in the level of HIV sentinel seroprevalence was observed immediately after the policy roll-out. The statistically significant natural growth rate showed an absolute year-to-year increase of 0.87%, indicating there would have been an annual rise of 0.87% on average of HIV sentinel seroprevalence had the treatment policy change not been implemented. The change in trend showed a significant absolute decrease of 3.08% of HIV sentinel seroprevalence on average each year since the policy implementation. Model 1 predicted that HIV sentinel seroprevalence among MSM in Chengdu would have reached 20.80% in 2018 without the intervention, while the actual figure was 5.17% for that year. Therefore, the absolute effect of Treat All policy was a significant drop of 15.63% in HIV seroprevalence, equating to a relative 75.14% decrease, four years after the treatment policy intervention.

According to Model 2 (Table 2), annual reported HIV cases had an average increase of about 116 cases per year during the pre-policy period, indicating 116 more cases would have been reported annually than the previous year had the treatment policy intervention not been implemented. A significant increase immediately after the policy implementation was observed, and we found a downward trend where there was a decrease of about 158 cases each year during the post-policy period, indicating on average some 158 fewer HIV cases were reported annually since the policy implementation. Model 2 estimated that annual reported HIV cases among MSM in Chengdu would have been 1344 cases in 2018 without the intervention, while the real number was 1014 cases that year. Therefore, the absolute effect of Treat All policy was a significant reduction by 330 newly reported HIV cases, a relative 24.55% decrease in other words, four years after the treatment policy intervention.

## Discussion

Our study showed HIV sentinel seroprevalence and annual reported HIV cases declined, and ART coverage rate increased after Treat All policy was implemented in MSM population in Chengdu, illuminating the potential of immediate ART initiation to contain the HIV epidemic. It also suggested that in real-world conditions, early ART could be potentially efficacious in curbing HIV transmission at population level, which were consistent with previous studies [48, 49].

Treat All policy contributed to reducing new HIV infections at population level among MSM and had a sustained impact. According to ITS analysis, compared to upward trends during the pre-policy period, there were statistically significant downward trends in HIV sentinel seroprevalence and annual reported HIV cases since the policy intervention, indicating the growing trends were reversed after Treat All policy implementation. Increased ART coverage is associated with decreased HIV incidence [13, 49, 50], and the huge progress made in ART coverage reflected Treat All policy helped ensure more efficient ART delivery and prompted more asymptomatic MSM living with HIV to undergo treatment, thus potentially turning down population-level HIV transmission, which were in line with similar real-world studies to show expanded access to ART helps curb the growth of HIV epidemics [48, 49].

The second “90” of UNAIDS’s “90–90–90” goals, which is 90% of those diagnosed with HIV receive ART, was achieved among MSM in this region. As is the case in this study, ART coverage rate reached 92.29% among MSM in 2018 (an absolute increase of 81.18% compared to that in 2008). After CD4+ cell threshold for ART eligibility was abolished, great progress has been made in terms of the proportion of HIV-positive MSM who’re receiving ART, which was also observed in previous study [13]. Scaling up ART coverage has been important component in actions against the growing HIV epidemic among MSM [30]. Increased ART coverage reflects direct effect of lowering the treatment eligibility threshold (CD4+ cell count), serving as the intermediate path from decreased threshold for ART eligibility to new HIV infection reduction. While real-world studies evaluating impacts of lowering treatment threshold on ART coverage and HIV epidemics were scarce, series of clinical trials concerning this aspect were carried out. Both the TasP (ANRS 12249) and the HPTN 071 (PopART) trials conducted in South Africa didn’t see increased ART coverage or reduced incidence in the immediate ART arm compared to the control group [27, 51]. The absence of decreased incidence can be explained by incident HIV infections from outside of the study population in light of multiple sex partners behavior among MSM [52], suggesting ART accessibility for “all” is critical in fighting against HIV. Besides, low treatment coverage in the trials added to the evidence of challenging situations where lack of funding, manpower and so on may emerge in trial settings, not to mention in real-world settings with more restricted budgets. Nevertheless, Treat All policy bore inspiring outcomes, which can be due to great work and robust funding from local and national authorities and organizations, plus budgets guarantee from China’s National Free Antiretroviral Treatment Program [53].

Lagged effect, referring to impacts taking time to manifest after intervention [54], wasn’t observed in our study, which can be due to that the HIV epidemic was in dire situation among MSM in Chengdu and MSM since has been the key intervention group, so that quick and great efforts were taken to roll out Treat All policy citywide. Besides, concurrent endeavors like expanded HIV testing and HIV/AIDS supportive environment construction may also play a part.

It should be noted that those recruited in the annual sentinel surveillance at venues frequented by MSM (like gay bars, bath room, etc.) are more likely to be sexually active with more risky sexual behaviors, thereby HIV sentinel seroprevalence may be somewhat overestimated [37]. But this influence isn’t prone to vary over time. Moreover, the proportion of previously HIV infected MSM in the surveyed sample has remained stable since the national HIV surveillance system scale-up in 2008, so HIV seroprevalence since 2008 can reasonably reflect changes in the trend of new HIV infections among MSM [37]. Hence HIV seroprevalence obtained from serial cross-sectional surveys could still reveal information about changing trends of the HIV epidemic and be used to evaluate effects of HIV prevention and control efforts among MSM.

Additionally, despite a descending trend since 2014, annual reported HIV cases among MSM remained at a relatively high level, compared with that in the pre-policy period. This may be attributed to the following aspects: optimization of national HIV information system making sure more complete and timely case reporting; increased number of MSM community-based organizations (CBOs) and their frequent activities prompting more hidden HIV-positive MSM to be detected; and rising number of HIV-positive MSM with an ongoing HIV epidemic in MSM population whose size is growing as well.

Our study reveals practical value of Treat All policy to potentially reduce HIV infection at population level, while challenges remain at the frontline. Treat All policy demands enormous investments to achieve effective ART expansion and sustained viral suppression, the ultimate goal of HIV control, which requires sustained ART accessibility. However, this goal is yet a challenge to be overcome financially and administratively, especially for low- and middle-income countries where resources of all sorts are limited [55]. Furthermore, due to social and structural factors like stigmatization, key populations affected by HIV including MSM have poor access to HIV services. But they are groups who benefit most from early ART [56]. Core-group theory posits that prevention among a relatively few at the highest risk of acquisition and transmission, can protect many along a potential transmission chain [57, 58]. Therefore, MSM have been and will still be a priority group in HIV prevention and control strategies globally.

To maximize the benefits of Treat All policy, experience can be learned from Chengdu and measures need to be taken from a systematic perspective. First of all, expand HIV testing in that it underlies implementation of nearly all other interventions. As supplement to voluntary counseling and testing, home-based “HIV self-testing” was suggested by WHO to improve HIV testing [59]. There are self-testing kits available now in Chengdu. Moreover, to encourage engagement, HIV testing services can be applied on CBO’s websites and provided in places frequented by MSM, which have helped improve testing volumes in Chengdu. Second, strengthen collaboration with international, governmental and non-governmental organizations to guarantee robust funding. Chengdu CDC worked with UNAIDS to successfully execute strategic planning of AIDS prevention and treatment for MSM (2011–2015) to facilitate field work and community empowering. Besides, during China’s “12th five-year plan on AIDS prevention and treatment”, Chengdu CDC collaborated with China CDC, the central institution in Beijing, to conduct questionnaire interviewing and serological testing among MSM. Finally, a Continuum of HIV Services should be established with CBOs, CDCs and hospitals coordinated as a whole, to improve health care efficiency, expand coverage and facilitate sustained accessibility. One-stop HIV testing, confirmation and treatment services are now available at the same hospital in Chengdu. The “Tongle” (“happy together”) organization, a Chengdu-based CBO which serves as a hinge to link MSM, local CDCs and hospitals, sets as a good example that support from peers and CBO workers trusted by MSM are necessary while delivering HIV services.

### Limitations

There are some limitations in this study. Firstly, HIV sentinel seroprevalence is obtained using non-probability sampling methods, which might lead to selection bias and undermine representativeness [39]. However, probability sampling is not practical considering the hidden nature of MSM population. The same sampling design used in each survey round guarantees any bias caused by nonrandom sampling can be assumed to be relatively stable [40], so samples of sub-groups of MSM still provide important information of the HIV epidemic as well as effects of policy intervention. Secondly, the number of time points used in ITS models was relatively small. Although there were only 6 and 5 time points before and after the policy intervention respectively, the analysis outcomes presented statistically significant trends, indicating robust statistical power to detect changes in the trends. Thirdly, viral suppression rate, the third UNAIDS “90” goal, is supposed to be taken into consideration in treatment policy evaluation. It wasn’t included due to data incompletion in the early period when the information system was being built up. Further research should work on evaluation of viral suppression to fully interpret impacts of HIV policy interventions. Finally, an observational study, our study was based on an ecological perspective, potential confounding might not be controlled. Any causal relationships can’t be assumed between the policy intervention and the analyzed indicators. Though ITS analysis didn’t control for other events that may have influenced the outcome, single group time series still address threats to internal validity and provide a methodologically acceptable design for studying intervention effects [54]. Besides, our study is limited in Chengdu, hence careful consideration and comparisons of geographical and subpopulation patterns of HIV epidemics among populations at provincial and national level should be taken before cautious generalization to broader populations.

## Conclusions

The policy of immediate ART after diagnosis in MSM population in Chengdu improved ART coverage, enabling more MSM living with HIV to receive treatment. And the downward trends of HIV sentinel seroprevalence and newly reported HIV cases demonstrated the potential of Treat All policy to turn the tide of the rampant HIV epidemic in MSM population. A real-world evaluation of treatment policy intervention among MSM, our study laid foundation for future assessment of HIV prevention and control policy using longitudinal routine data from health information systems, particularly in resource-limited low- and middle-income countries. In the meanwhile, further coordinated strategies are needed to maximize the benefits of early ART to contain HIV epidemics.

## Data Availability

The data that support the findings of this study are not publicly available due to data protection and confidentiality and restrictions apply to the availability of these data, which were used under license for the current study. Data are however available from the authors upon reasonable request and with permission of Chengdu CDC.

## Abbreviations

ART: Antiretroviral therapy/treatment
AIDS: Acquired immunodeficiency syndrome
APC: Annual Percent Change
AAPC: Average Annual Percent Change
CDC: Center for Disease Control and Prevention
CRIMS: National HIV/AIDS Comprehensive Response Information Management System
CI: Confidence interval
HIV: Human immunodeficiency virus
ITS: Interrupted time series
MSM: Men who have sex with men
PLHIV: People living with HIV
UNAIDS: The Joint United Nations Program on HIV and AIDS
WHO: World Health Organization
